# Conflict Resolution Ability in Late Bilinguals Improves With Increased Second-Language Proficiency: ANT Evidence

**DOI:** 10.3389/fpsyg.2019.02825

**Published:** 2019-12-20

**Authors:** Nikolay Novitskiy, Yury Shtyrov, Andriy Myachykov

**Affiliations:** ^1^Department of Linguistics and Modern Languages, The Chinese University of Hong Kong, Shatin, China; ^2^Brain and Mind Institute, The Chinese University of Hong Kong, Shatin, China; ^3^Centre for Cognition and Decision Making, National Research University Higher School of Economics, Moscow, Russia; ^4^Department of Clinical Medicine, Center of Functionally Integrative Neuroscience, Aarhus University, Aarhus, Denmark; ^5^Laboratory of Behavioural Neurodynamics, Saint Petersburg State University, St. Petersburg, Russia; ^6^Department of Psychology, Northumbria University, Newcastle upon Tyne, United Kingdom

**Keywords:** bilingualism, second-language learning, cognitive control, bilingual advantage, Russian language

## Abstract

Experimental data supporting the claim that bilingual speakers have superior cognitive control abilities are often questioned with respect to certain methodological limitations. One such limitation is the use of between-group design, potentially confounding bilingual status with other factors (e.g., socioeconomic status). Here, we used a homogeneous sample of 57 young adult Russian–English late unbalanced bilinguals who were administrated Attention Network Task (ANT) together with an L2 proficiency task. We tested the correlation of L2 vocabulary performance with conflict and alertness measures and overall reaction times in ANT performance. Overall, participants demonstrated better conflict resolution with the increase in their second language competence, with 8% of variance in conflict resolution explained by L2 proficiency. Our results support the notion of regular correspondence between bilingualism and cognitive control.

## Introduction

Bilingualism – the ability to speak two languages – is ubiquitous among the world’s population. Current estimates suggest that over a half of Earth population are bilingual or multilingual (Bialystok et al., 2012). Living with two languages can be considered not only a challenge but also an opportunity and a benefit. This latter view is reflected in existing literature, most notably, suggesting that bilingualism is associated with a more efficient cognitive control function, both in language-related and language-unrelated task performance (Kroll and Bialystok, 2013; Costa and Sebastián-Gallés, 2014; Bialystok, 2015).

Cognitive control covers a number of distinct processes involved in goal-oriented behavior (Miyake and Friedman, 2012). These include monitoring current task-related activities, inhibiting irrelevant actions and task switching (Miyake et al., 2000; Miyake and Friedman, 2012). Cognitive control is traditionally closely associated with the brain’s attentional system that includes three distinct networks: those for alerting, orienting, and executive control (Posner and Petersen, 1990; Petersen and Posner, 2012). It is often assumed that attention is a domain-general function; i.e., the same brain resources are used in different (visual, auditory, verbal etc.) tasks (Corbetta and Shulman, 2002; Corbetta et al., 2008).

Regular use of two or more languages by the same person requires frequent switching between the languages as well as the need to inhibit input and output in the irrelevant language when a currently relevant language is selected (Abutalebi, 2008; Green and Abutalebi, 2013). Therefore, this feature of bilingual processing should support the development of a stronger cognitive control system, allowing bilinguals to switch efficiently and rapidly between languages. Indeed, bilinguals have often been shown to outperform monolinguals on cognitive control tasks both in accuracy and reaction times (RTs), even when the task itself is non-verbal and does not directly imply any linguistic processing (Kroll and Bialystok, 2013; Costa and Sebastián-Gallés, 2014; Bialystok, 2015). This has been demonstrated using a variety of behavioral tasks including Simon (Bialystok et al., 2004), flanker (Costa et al., 2009), and task switching paradigms (Prior and Macwhinney, 2010). This “bilingual advantage” effect has been found across different age groups: in children (Bialystok and Martin, 2004), young (Costa et al., 2008), and older adults (Gold et al., 2013).

Essentially, enhanced cognitive control in bilinguals may result from brain rewiring (Abutalebi et al., 2012; Luk et al., 2012; Green and Abutalebi, 2013): As the two languages regularly tap into the same domain-general resources, selecting between them requires more frequent recruitment of control systems, eventually leading to performance enhancement. The original hypothesis put forward by Bialystok and colleagues specifically suggests enhancement of inhibition abilities (e.g., when filtering out distracting information, Bialystok, 1999). Other data, however, support a general performance improvement even in situations when inhibition *per se* is not required (Bialystok et al., 2004). In addition, the bilingual advantage effects seem to be stronger in children and older adults (Bialystok et al., 2005, 2008; Lee Salvatierra and Rosselli, 2011). For example, bilingualism was even suggested to delay the Alzheimer’s disease symptoms onset, although it does not prevent its occurrence (Bialystok et al., 2007, 2016). Bilingual advantage in children, at least hypothetically, could result from a larger degree of the cortical malleability; however, it is unlikely that the effect suddenly disappears in young adults to reappears again in senior age. More plausible explanations attribute the lack of differences between monolingual and bilingual young adults to a ceiling effect due to the optimal development of cognitive control system in this age group, i.e., the tasks are simply too easy for both monolingual and bilingual young adults to show any measurable difference in performance (Gold et al., 2013).

Regardless of the abundant evidence in favor of the bilingual advantage, this notion is often criticized for certain methodological caveats, e.g., the objectivity of participant assignment to groups and other potential confounds (Paap and Greenberg, 2013). Demonstrations of bilingual advantage effect traditionally employ a group design comparing performance of bilingual participants to that of their monolingual peers. However, bilingual and monolingual speakers may differ not only in the number of languages they regularly speak but also in other aspects including their socioeconomic status (SES) as the former are often recruited among immigrants and ethnic minorities. Although immigrant status itself is irrelevant to the cognitive and linguistic abilities of a participant, there are reasons to consider immigrants as atypical group in relation to both their host and original populations. It should thus be beneficial to test the connection between cognitive control and proficiency in languages within a single homogeneous sample rather than comparing monolingual and bilingual groups. To that end, here, we use language proficiency and cognitive control task performance as two continuous variables to measure potential correspondences between them (see below).

Furthermore, a number of studies have been unsuccessful in replicating the effect. For example, comparisons of large children samples from different areas of Spain, bilingual Basque Country, and monolingual areas showed a null effect (Antón et al., 2014). A similar null result was reported in a study comparing elderly populations in the same areas (Antón et al., 2016). In both studies, the mono- and bilingual subject samples were thoroughly matched in age, gender, IQ, education, and SES. It is also noteworthy that bilingual advantage effect is often registered in smaller sample studies, while studies using bigger samples often yield negative results (Paap et al., 2015). It has been suggested that at least some positive results in the bilingual advantage literature reflect a publication bias, i.e., the negative results are withdrawn by authors or prevented from being published by reviewers and editors on the grounds of insufficient statistical power (de Bruin et al., 2015). Some critics of the bilingual advantage effect go as far as to claim that it is merely a methodological artifact or, at best, it is limited to very specific population groups (Paap et al., 2015, 2016).

Nevertheless, Costa et al. (2008) provided one of the most comprehensive demonstrations of the bilingualism advantage with a large sample size (*N* = 100 per group). Young adult Spanish–Catalan balanced bilinguals and Spanish monolinguals performed on a version of Attention Network Task (ANT) (Fan et al., 2002). ANT is a combination of cued RT tasks (Posner, 1980) and a flanker task (Eriksen and Eriksen, 1974). The results showed a clear advantage of bilinguals over monolinguals in several measurements. First, bilinguals’ RTs were *overall* faster than those of monolinguals. Second, the RT performance difference between congruent and incongruent trials was *smaller* in bilinguals indicating better conflict resolution. Third, *orienting* performance was associated with larger RT delays in bilinguals than in monolinguals. Fourth, bilinguals showed a smaller RT cost for switching between different types of trials, especially for the more difficult switch from incongruent to congruent trials. Overall, this pattern generally supports the notion of a stronger and a more efficient cognitive control system in early bilinguals. Given that these results were obtained in balanced Spanish-Catalan bilinguals, they also pose a question whether similar positive effects may emerge for late unbalanced bilinguals when the two languages are not used equally often on a day-to-day basis (Costa et al., 2008).

Indeed, early balanced bilinguals are a rather rare group outside few areas with historic preponderance for multilingual environment. As mentioned above, this type of bilingualism is not easily comparable to a more typical bilingualism when the second language is acquired later in life and where the resulting language proficiency is not balanced between L1 and L2. Furthermore, even early immersion in another language environment, as in the case of immigrant children or heritage speakers, does not necessarily lead to an efficient bilingualism as evident in the abundance of L1 attrition cases (Pallier et al., 2003). Finally, bilingualism is not a categorical phenomenon; instead, it can be viewed as a continuum characterized by a differentially graded balance between individual’s L1 and L2 proficiencies. Globalization and the increase in Internet use have promoted English to the position of a *de facto* international *lingua franca* (Piercy, 2012). As a result, the population of late English-local bilinguals becomes arguably one of the largest bilingual groups, and its study is therefore associated with substantial practical importance. In other words, this type of bilinguals – characterized by relatively late L2 acquisition and the absence of full balance between L1 and L2 – provides a much more typical population sample than purpose-selected immigrant groups or populations of ethnolinguistic enclaves. What is even more important in the current context is that this population provides a gradual measure of L2 proficiency, which can then be linked to executive task performance in a within-group (rather than between-group) design.

One recent study (Xie, 2018) examined cognitive control using a version of the Flanker task in a sample of unbalanced Chinese–L1 bilinguals with English as their L2 recruited in their home country using a version of the Flanker task. While the data did not reveal any difference in the inhibition cost, an overall faster RT in the most proficient bilinguals was observed as compared to the least proficient ones. These findings, together with the factors discussed above, motivated our choice of the design for the study reported below.

Here, we tested cognitive control performance in a group of late unbalanced Russian–English bilinguals. To circumvent some of the problems related to participant selection, we selected a homogeneous group of bilingual participants with native Russian L1 and proficient but variable English L2. To test their L2 skills and quantify it as a graded variable numerically, we assessed their proficiency using established tests and a custom-made L2–L1 vocabulary test using a varied set of words with a wide range of corpus lexical frequency. Although there is currently no “golden standard” test for the bilingual proficiency, vocabulary tests are the easiest to be quantified (for review, see Treffers-Daller, 2019). To justify the relevance of English corpus frequency to our bilingual participants’ vocabulary, we tested the relation between item frequency, familiarity, and speed of recognition (Burgess and Livesay, 1998). To measure participants’ executive control function in a similar way, we used the ANT, an established measure used to assess different aspects of the cognitive control mechanism (Fan et al., 2002). To assess the putative links between the executive processes and the participants’ bilingual status, we built a regression model of conflict cost as a function of participants’ performance in the L2–L1 language task. Following the bilingual advantage studies reviewed above (e.g., Costa et al., 2008), we hypothesized that the conflict control in young adult late unbalanced bilinguals will increase with their L2 proficiency. If bilingualism, as a consequence of L2 acquisition and use, leads to the enhancement of executive control system, then the conflict cost should decrease with increasing L2 proficiency and vocabulary size. Alternatively, if the bilingual advantage is an artifact of unbalanced between-group comparisons, we could expect to find no such effects in our homogeneous sample of participant.

## Materials and Methods

### Experimental Participants

Fifty-seven Russian–English bilingual participants (19 male) were recruited from the student population at the Higher School of Economics, Moscow. Their median age was 19, mean age of 20.6 ± 0.51 years. Right-handedness was a requirement for participation. Participants were presented with the tests in the following order: (1) The Language Experience and Proficiency Questionnaire (LEAP-Q) test (Marian et al., 2007), (2) L2 vocabulary test, and (3) ANT test (version 1.3.0). The participants that made more than 30% of errors in vocabulary test were excluded from the analysis. The study was performed in accordance with the Helsinki Declaration and approved^1^ by the local Research Ethics Committee; written consent was obtained from all participants.

### Subjective Language Proficiency and Exposure

Subjective L2 proficiency and exposure were measured with the help of the LEAP-Q (Marian et al., 2007) implemented in NBS Presentation 18.1 stimulus presentation software (Neurobehavioral Systems, Berkeley, CA, United States) such that each question in the questionnaire appeared as one computer screen to the participant. English proficiency also featured as one of the questions, and it has to be self-estimated by participants separately for speaking, understanding, and reading on a scale from 1 to 10. The average of speaking, understanding, and reading scores was used as a combined measure of English proficiency, hereafter referred to as subjective proficiency.

### Vocabulary Test

**FIGURE 1:**
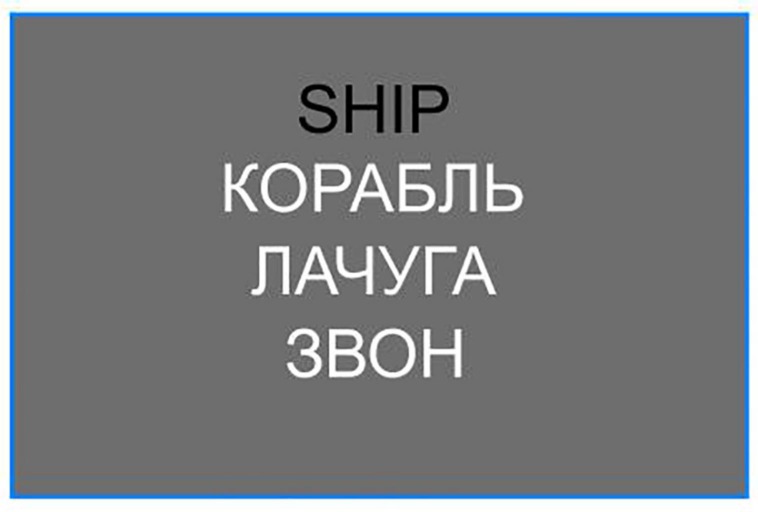
Vocabulary test slide example. The participant chose correct translation by pressing keyboard buttons 1, 2, or 3. In this example the correct answer is 1 (“КОРАБЛЬ” = “ship”). The distractors are “shack” (ЛАЧУГА) and “toll” (ЗВОН).

Objective L2 proficiency was tested with a custom designed vocabulary test, which was created with a goal of providing a numerical measure of participants’ L2 vocabulary. One hundred forty-six English monosyllabic words were selected from the COCA (Corpus of Contemporary American English, 2016) online database. To ensure a wide range of vocabulary, lexical frequency of occurrence of the base forms and all inflections of these items (Lemma Frequency) varied between 0.71 and 566.99 ipm (mean, 61.59 ipm). Vocabulary test was a three-interval forced choice unspeeded task implemented in NBS Presentation 18.1 stimulus presentation software. Every participant was presented with an English word (black font on gray background) and three translation versions in Russian (white font on gray), all in one column (see Figure 1). Participants had to choose the correct translation by pressing 1, 2, or 3 on the keyboard. Feedback was provided immediately after response for both correct (in green font) and incorrect (in red font) answers. The number of incorrect responses was used as a measure of participant’s proficiency in L2, hereafter referred to as Vocabulary Errors. In addition to the participant-wise data, the item-wise RTs were extracted, hereafter referred to as Item RT and item-wise proportion of competent participants, hereafter referred as Item Accuracy.

### ANT Test

**FIGURE 2:**
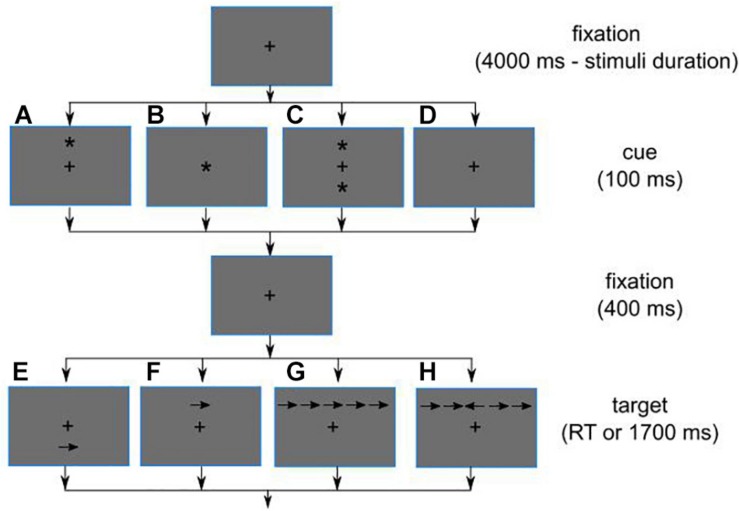
The schematic diagram of trial sequence in Attention Network Task (ANT). The fixation is followed by the cue that primes one of two peripheral location **(A)**, central location **(B)**, both peripheral locations **(C)**, or no location **(D)**. The interim fixation is followed by single **(D,E)** or flankered target **(F,H)**. The cue **(A)** provides orientation cueing, the cues **(B,C)** increase alertness. The target can be incongruent **(D)** or congruent **(E–H)** in respect to the orientation cue **(A)**. The target **(G)** provides no-conflict (congruent) condition, while the target **(H)** embeds a conflict in arrow orientation. Note that some slide deducible configurations are not shown, i.e., orienting cue as well as flankered targets can be positioned at both spatial locations.

The Java version of ANT was downloaded from the ANT developers’ website (Fan, 2016). First, the instructions appeared on the screen, and the participants were advised to read them silently. The instructions directed the participant to detect the horizontal direction of the arrow pointing on the screen and report it by pressing the corresponding arrow key. The arrow was presented above or below the fixation point. In *conflict* condition, the arrow was flankered by congruent or incongruent distractor arrows, two on each side. In *orienting* condition, the target was cued by an asterisk at congruent or incongruent location. Alternatively, the cue could be presented at both locations (*alerting* condition), at the place of the fixation or not presented at all. The duration of the cue screen was 100 ms. It was separated from the target by a 400-ms fixation interval. The target screen was presented until the participant responded or 1,700 ms, whichever took shorter. The duration of each trial was on average 4 s, and the fixation cross was always on the screen except for cue and target presentations (Figure 2).

The experiment started with a training block of 14 trials, followed by three experimental blocks of 96 trials each. The trials included all combinations of experimental conditions and followed each other in individually randomized order and in a fully counterbalanced fashion. In relation to the cue, this means that each of the no-cue, fixation location cue, and both locations cue trials occurred 24 out of 96 of total trials (25% of the time) and that there were 12 of the upper and lower cue trials. In relation to the target, it was pointing right or left in 48 trials out of 96 (50%) of the time and was accompanied by flankers in 64 trials with half of them pointing in the same or different direction (congruent and incongruent trials correspondingly).

Accuracy and RTs were analyzed according to the standard ANT protocol, producing a set of parameters for each participant: (1) alerting effect, defined as the difference in RT between non-cued and cued conditions; (2) orienting effect (difference in RT between congruent and incongruent cued conditions), (3) conflict effect (difference in RT between congruent and incongruent distractor conditions), (4) mean RT, and (5) mean accuracy. We normalized the conflict, alertness, and orienting effects by dividing them by the individual overall RT and multiplying by 100%. The latter estimates were used in regression models as conflict effect rate, alertness effect rate, and orienting effect rate correspondingly.

### SES and Intelligence

In addition, participants perform on an online test of intelligence and SES. The tests were programed in Google Forms, and the participants were provided with a metalink. The intelligence test included Raven matrices (Raven, 1941). The original Raven matrix set consists of 5 series of 12 matrices. The series are labeled with Latin letters A–E with their complexity increasing from A to E. We used 10 matrices: C9, C10, E2, E4, E5, E6, E7, E8, E9, and E12. Each picture in the Raven matrix task consisted of a drawing of nine components including a blank component in the lower right corner of the drawing. There was an implicit rule in the drawing, and the task of the participant was to fill the blank space by choosing one of the eight drawing segments placed under the main drawing. The percentage correct was transformed to IQ values.

SES measure was developed from the sociodemographic questionnaire by MacArthur Network^2^. We have chosen the shortened version with eight questions translated into Russian with income categories adjusted according to the distribution of income in Russia as provided by the Federal Service of State Statistics^3^. Among the participants who filled in the SES test, only 30 (71%) and 28 (67%) responded to the questions about their individual and family incomes correspondingly, for privacy reasons. In total, 43 participants filled in the intelligence test and 42 performed the SES test.

### Statistical Analysis

Statistical analyses were performed in MATLAB with the help of Statistics and Machine Learning Toolbox (MathWorks, Natick, MA, United States). The Gramm toolbox was used for plotting (Morel, 2018). The following regression models were tested: (1) subjective proficiency vs. vocabulary error rate, (2) log lemma frequency vs. item accuracy, (3) log lemma frequency vs. item RT, (4) conflict effect rate vs. vocabulary error rate, (5) alertness effect rate vs. vocabulary error rate, and (6) overall RT vs. error rate. For plotting purpose only, Eilers’ smoothing has been applied to the data points on the figures (Eilers, 2003). Means ± SEM for all variables are reported as well as adjusted *r*^2^, regression coefficients β, *p* values for each regression analysis are reported.

In addition, we applied stepwise regression to select the measures that would improve our original two-variable approach. To that end, we collected all available information into one matrix and run the MATLAB function *stepwise* that automatically adds or removes terms into/from the multiple regression model on the basis of the *F* test comparison between the updated and current model. Our original model was formulated as *conflict*∼1 + *vocabulary error rate*, and the list of potential term to add included mean RT, mean accuracy, alertness effect rate, orienting effect rate, subjective proficiency, education level, IQ, age, sex, number of learned languages, age of onset of English speaking, and age of onset of English reading.

## Results

Table 1 provides the summary of the LEAP-Q results for Russian and English. Russian was learned significantly earlier than English; therefore, the participants’ proficiency in the former was higher than in the latter. The participants were exposed more to Russian than English in their communication with friends and especially family as well as while watching TV, but not while listening to music or reading (including reading online). There was a tendency for more exposure to English than Russian music.

**TABLE 1:** The results of Language Experience and Proficiency Questionnaire (LEAP-Q) questionnaire for Russian and English for 57 subjects.

	**Russian**		**English**		
	**Mean**	**SD**	**Mean**	**SD**	
**Critical age (years)**					
Start speaking	0.4	0.87	7.4	2.68	^∗∗∗^
Fluent speaker	3.8	2.09	13.8	4.04	^∗∗∗^
Start reading	4.6	1.19	9.3	3.22	^∗∗∗^
Fluent reader	6.4	1.86	13	4.13	^∗∗∗^
**Language environment (years)**					
Country	20	4.52	0.6	1.45	^∗∗∗^
Family	20.5	3.76	0.5	2.59	^∗∗∗^
School/work	16.9	5.27	1.7	3.06	^∗∗∗^
**Proficiency, 0–10**					
Speaking	9.8	0.54	7.3	1.49	^∗∗∗^
Understanding	10	0.13	7.7	1.48	^∗∗∗^
Writing	9.9	0.43	8.3	1.4	^∗∗∗^
**Contributing_factors, 0–10**					
Friends	8	1.96	4.5	3.29	^∗∗∗^
Family	9.3	1.04	1.3	2.47	^∗∗∗^
Reading	8.9	1.87	8.2	1.64	–
TV	5.6	3.26	3.9	3.42	^∗^
Music	5.7	3.11	7	2.3	–
**Exposure, 0–10**					
Friends	9.4	1	3.2	2.73	^∗∗∗^
Family	9.7	0.99	0.6	1.47	^∗∗∗^
Reading	8.2	1.85	7.1	2.19	–
TV	6.1	4.24	3.2	3.37	^∗∗^
Music	5.7	3.23	6.9	2.62	–
**Accent, 0–10**					
Self estimate	0.5	1.4	5.1	2.4	^∗∗∗^
Others’ estimate	0.4	1.26	7	3.14	^∗∗∗^

*The asterisks indicate significant differences between English and Russian (^∗∗∗^*p* < 0.001, Bonferroni corrected).*

Most of the participants (50 out of 57) reported some knowledge of the languages other than Russian and English. Altogether, 26 different languages were named; the most popular among them were German (22 participants), French (20 participants), and Spanish (15 participants).

**FIGURE 3:**
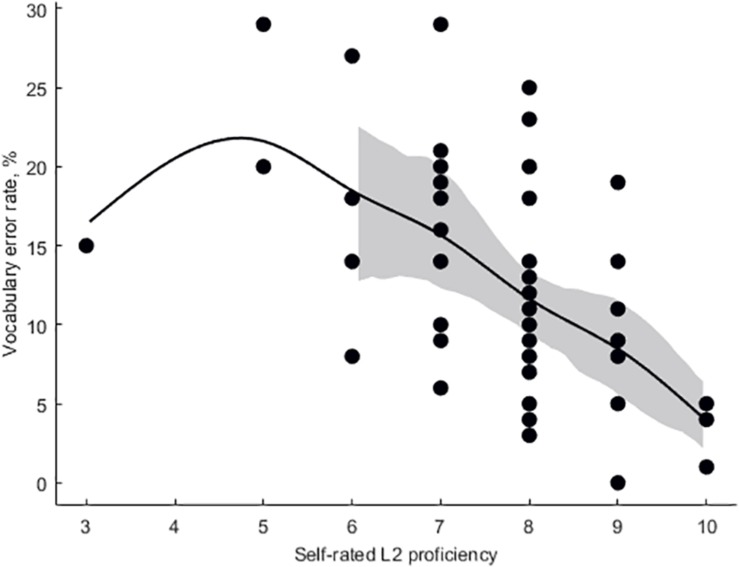
Error rate in vocabulary task a function of self-rated English proficiency (Eilers smoothing).

**FIGURE 4:**
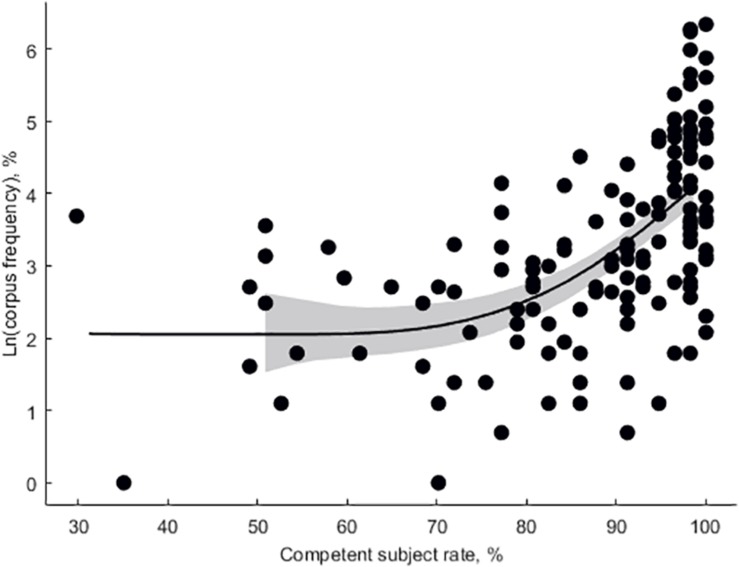
The log-transformed item corpus frequency as a function of the competent participants number per item (Eilers smoothing).

**FIGURE 5:**
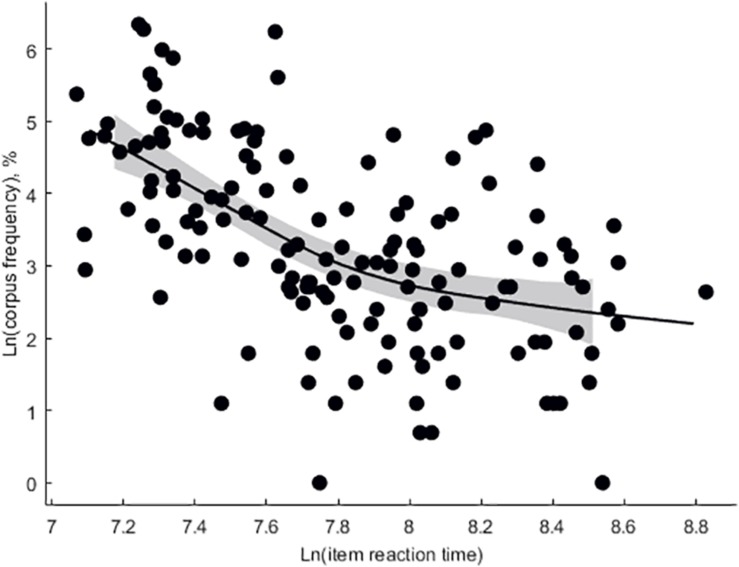
The log-transformed item corpus frequency as a function of the log-transformed item reaction time in the vocabulary task (Eilers smoothing).

**FIGURE 6:**
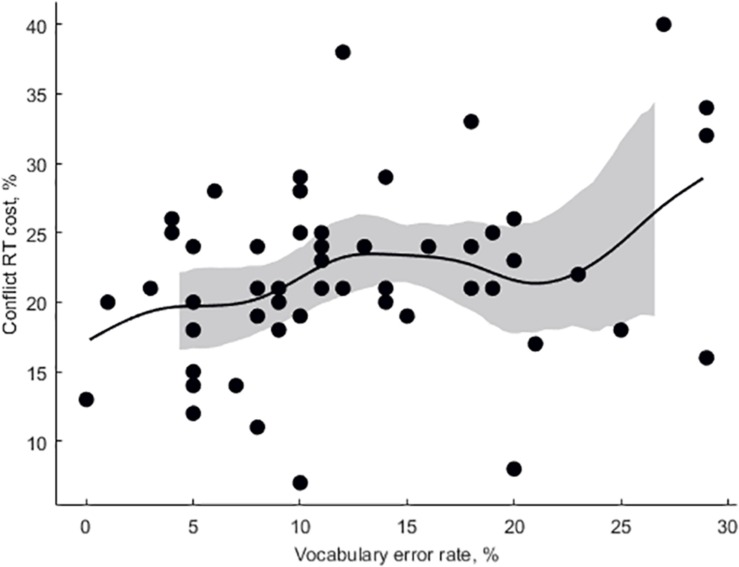
The percent of conflict effect rate from the Attention Network Task (ANT) as a function of error rate in vocabulary proficiency task (Eilers smoothing).

Mean participant-wise error rate in vocabulary task was 12.5 ± 0.96%. Mean item-wise RT in vocabulary task was 2,651 ± 95 ms. Mean RT in ANT was 591 ± 10.4 ms, mean alertness effect rate was 5.6 ± 0.53%, and mean conflict effect rate was 22 ± 0.84%. Subjective proficiency negatively correlated with vocabulary error rate (adjusted *r*^2^ = 0.27, β = −0.097, *p* < 0.001, Figure 3). Log lemma frequency positively correlated item accuracy (adjusted *r*^2^ = 0.25, β = 5.49, *p* < 0.001, Figure 4). Log lemma frequency negatively correlated log item RT (adjusted *r*^2^ = 0.30, β = −0.17, *p* < 0.001, Figure 5). Most importantly, conflict effect rate positively correlated with vocabulary error rate (adjusted *r*^2^ = 0.08, β = 0.36, *p* = 0.0159, Figure 6). No correlations either between alertness effect rate and vocabulary error rate (adjusted *r*^2^ = 0, β = −0.06, *p* = 0.799) or between overall RT and vocabulary error rate (adjusted *r*^2^ = 0, β = 0.01, *p* = 0.413) were found. The global status of the participants was 6 ± 0.23 (out of 10), local status was 6 ± 0.28 (out of 10), individual income level was 2 ± 0.16 (out of 4), and family income level was 3 ± 0.13 (out of 4). The IQ was 111 ± 3.8. None of the SES or intelligence measures correlated with conflict effect rate. Vocabulary error rate correlated negatively with individual income level (adjusted *r*^2^ = 0.24, β = −4.27, *p* = 0.003). No other SES or intelligence measures correlated with vocabulary error rate.

The only variable that has been added by the stepwise algorithm was the mean accuracy. It improved the original model with *F* = 4.63 and *p* = 0.036. Note that the term vocabulary error rate was not excluded from the model. The final model took a form

ConflictCost∼1+vocabularyerrorrate+meanaccuracy

It explained 14.2% variance in conflict (adjusted *r*^2^). Within the model, the estimate (β) of the intercept was 91.3 (SE = 33.9, *t* = 2.69, *p* = 0.0093), of the vocabulary error rate was 0.28 (SE = 0.11, *t* = 2.59, *p* = 0.012), and of the hit rate was −0.75 (SE = 0.35, *t* = −2.15, *p* = 0.0358). In other words, the multiple regression not only confirmed the positive correlation between the conflict cost and number of errors in the vocabulary task but also showed lower hit rate for the participants with larger conflict costs, thus excluding the possibility of the accuracy–time trade of in the ANT.

## Discussion

The current study tested a group of late unbalanced bilinguals on their performance on a version of ANT and a translation task with a set of L2 words with a wide range of corpus frequency. Most importantly, relative increase in L2 vocabulary proficiency reduced the conflict cost in ANT performance in a within-group study, which makes a substantial contribution to the research in bilingualism effect on the cognitive control functions in young adult unbalanced late bilinguals (Tao et al., 2011; Singh and Mishra, 2013; Poarch et al., 2014; Singh et al., 2015; Vega-Mendoza et al., 2015; Mishra et al., 2018; Xie, 2018; Bonfieni et al., 2019).

Still, the relationship between bilingualism and cognitive control remains controversial (Hilchey and Klein, 2011; Paap and Greenberg, 2013). Many studies demonstrated improved cognitive control function in bilinguals, often referred to as bilingual advantage (Bialystok, 1999; Bialystok et al., 2004; Costa et al., 2008; Prior and Macwhinney, 2010; Gold et al., 2013), but this notion has recently received a great deal of criticism for what is portrayed as methodological drawbacks (de Bruin et al., 2015; Paap et al., 2015). Indeed, a recent meta-analysis revealed a small bilingual advantage for inhibition, which disappeared after the correction for publication bias (Lehtonen et al., 2018). In his balanced review, Antoniou tries to find the reason for the seemingly contradictory results and concludes that more studies on the multilingual experience are urgently needed (Antoniou, 2019).

Importantly, many studies on bilingual advantage are conducted as group comparisons between bilingual and monolingual participants. It had been argued that group differences could arise from a number of other factors than bilingualism, most importantly SES, education, and immigrant status. It is also worth noting that bilingualism in population is often a continuous rather than categorical variable. On the one hand, bilinguals are seldomly equally exposed to their languages, and as a result, one of the two languages is typically dominant; it is usually L1, but it can be L2 as well as in case of L1 attrition (Pallier et al., 2003). On the other hand, foreign language is a core subject in almost every school curriculum. Together with increasing travel affordability, massive migration, and the rise in global digital media expose, this leads to the establishment of a large effectively bilingual population segments even in traditionally monolingual communities. Thus, comparing monolinguals and bilinguals as two distinct groups assesses only the extreme ends of the bilingualism continuum and runs the risks of overlooking the confounding group differences.

Our study effectively overcomes the aforementioned limitations by comparing L2 proficiency and cognitive control abilities within a homogeneous group of young adult unbalanced late bilinguals. To that end, we used a group of native Russian students of similar SES that all share a similar history of acquiring English as their L2. They studied it at school and in the university as part of the syllabus and further practiced it during their studies (English-based tuition provided by the university) as well as for social reasons (e.g., for traveling abroad). Homogeneity of educational level and socioeconomic situation is a direct consequence of their status as students of one of the most prestigious universities in Russia located in the capital. Living and studying in Moscow, a large cosmopolitan city with a big expat population where English is often used in communication, in the media, and on the internet, makes these participants highly representative of the country’s growing group of young unbalanced bilinguals. Although not all of them are natives of Moscow, all were L1 Russian speakers originating from Russia/USSR, and none of them were immigrants.

This sample is also representative of the population of young educated Russians overall, and its use in our investigation may have important social implications. Late bilinguals worldwide are more common than early balanced bilinguals, but they remain understudied in relation to the development of their cognitive functions. Therefore, our study is an important step toward a better understanding of L2 learning and cognitive control relation. Importantly, while our results identified links between L2 proficiency and bilingualism, no such connection was found for IQ, which rules out the possibility that the effects are driven by general intelligence differences within the group.

While young adults are the most commonly studied population group in psychology, they are also the most difficult one in the bilingual advantage research, as the advantage effects are most elusive in this age group (Bialystok et al., 2008, 2005; Lee Salvatierra and Rosselli, 2011). The proponents of bilingual advantage concept often argue that it manifests better in children and elderly people and interpret the negative results in young adults in terms of a ceiling effect, as this is the age group on the peak of their cognitive control system development and therefore may have little space for bilingualism-related improvement (Gold et al., 2013). Thus, the current finding of bilingual advantage confirmation in young adults gives a strong confirmation to the concept.

We used a well-established measure of executive control in the form of ANT (Fan et al., 2002). This test provides a fast and efficient estimate for three main executive/attention mechanisms (Petersen and Posner, 2012) with executive control measured by conflict cost in RT. An earlier large-sample group comparison study demonstrated that balanced bilinguals show a reduced conflict costs in ANT (Costa et al., 2008). This evidence provided us with a clear-defined hypothesis for our within-group study, namely, that the conflict cost for our L1-Russian participants would be larger in those participants that committed more errors in the English translation task. The hypothesis was confirmed by linear regression analysis, which indicated a significant link between the ANT test results and L2 proficiency.

Our L2 vocabulary task included items with different corpus frequency that was supposed to provide more variability to our participant performance. Indeed, our data confirmed that the higher lexical frequency of an item improved its recognition and decreased the RT among participants, as it is expected from monolingual studies (Burgess and Livesay, 1998). It underlines that the presence of errors in the task is not due to the confounding factors, such as general fatigue and experimental environment, but reflect genuine language proficiency in English. This was also confirmed by strong correlation between vocabulary proficiency and the subjective self-reported proficiency that varied from very high to upper-middle.

Still, there are certain limitations to our study that need to be overcome in future research. Some of our participants reported knowing languages other than English and Russian. At least one recent study has indicated that the number of languages spoken by an individual has a positive effect on their learning capacities (Kimppa et al., 2016), calling for future investigations of the effects of L2 as well as L3, etc. on general cognitive abilities.

Another word of caution is with regard to the fact that the correlation between high vocabulary proficiency and efficient conflict resolution does not directly imply either the presence of a causal link or the direction of causation if it indeed takes place. While mastering another language can enhance one’s cognitive control, the opposite might be true as well, and the early development of cognitive control skills can in principle improve one’s proclivity for languages. Finally, there could be other factors driving both these changes. As such, while our research confirms the existence of an interplay between L2 proficiency and cognitive control system, it does not allow to favor any of the causal possibilities mentioned above. However, the very presence of the observed correlation can be tentatively and very cautiously interpreted as the effect of bilingualism on cognitive control development. According to the adaptive control hypothesis (Green and Abutalebi, 2013), the knowledge and usage of another language requires constant switching and monitoring of one’s verbal behavior, thus providing essential training to the cognitive control system in the prefrontal cortex and basal ganglia.

Finally, while a similar research question has been recently addressed by another study (Xie, 2018), there are important differences between this study and the one reported here – both in terms of the design and in terms of the reported findings. First, our study revealed a correlation between the conflict cost in the ANT (i.e., Flanker) task and the number of errors in the vocabulary translation task that was our measure of proficiency. In contrast, no difference in the inhibition cost between the groups of the bilinguals with different proficiency was found in Xie’s experiments. They found, however, an overall faster RT in the most proficient bilinguals as compared to the least proficient ones. Several important differences in the studies’ design can explain that seeming contradiction. First, the measure of proficiency was different in the two studies. We employed a visually presented written translation task with multiple choice, while Xie used a category L2 verbal fluency task. Although both tasks eventually tap the same property, i.e., proficiency, the difference in the implementation may introduce subtle differences that will make one measure closer to the dynamics of the cognitive control than the other measure. In our study, the stimuli in both cognitive control and proficiency were presented in visual domain, while Xie was looking for correspondence between the visual Flanker task and auditory verbal fluency task. Our task focused on perception of the linguistic stimuli, while their task measured production. The task in Xie’s study was fully in L2, while our task included translation, i.e., language switching. The previous work of Dong and Xie has demonstrated that switching experience can have more intimate link with the cognitive control task than pure proficiency (Dong and Xie, 2014). Perhaps, adding the element of switching to our task made it more sensitive to those differences in proficiency that is relevant for cognitive task performance. Second, Xie used group design with an arbitrary split of the subject into high-, middle-, and low-proficiency groups. Our study treated both cognitive control performance and L2 proficiency as continuous variables. Those two factors, different proficiency test, and group vs. continuous design may have resulted in the different outcomes in the two studies. However, while different in the details, our findings essentially prove the same thing, namely, that the difference within bilingual groups in, among other things, their L2 proficiency can lead to the difference in their cognitive control performance.

## Conclusion

In conclusion, young adult late unbalanced bilinguals in our study demonstrated better conflict resolution with the increase in their second language competence. This correlation supports the concept of the beneficial effect of bilingualism, for the first time, showing it within a group of late unbalanced bilinguals rather than across groups. The results may have implication for the theory of cognitive control and language interaction in the brain as well as for educational practices.

## Data Availability Statement

The datasets generated for this study are available on request to the corresponding author.

## Ethics Statement

The studies involving human participants were reviewed and approved by the Higher School of Economics Research Ethics Committee, Russia. The patients/participants provided their written informed consent to participate in this study.

## Author Contributions

NN performed the data collection and analysis, formulated the hypothesis, and wrote the manuscript. YS and AM contributed to the reviewing, editing, hypothesis, and supervision of the manuscript.

## Conflict of Interest

The authors declare that the research was conducted in the absence of any commercial or financial relationships that could be construed as a potential conflict of interest.

## Footnotes

1 https://www.who.int/bulletin/archives/79(4)373.pdf

2 https://macses.ucsf.edu/research/socialenviron/sociodemographic.php

3 www.gks.ru
